# Meal‐Induced NF‐kB Activation in Mononuclear Cells Triggers Lumican Secretion and Promotes Chronic Kidney Disease in Metabolic Dysfunction‐Associated Steatotic Liver Disease

**DOI:** 10.1002/mco2.70122

**Published:** 2025-08-07

**Authors:** Giovanni Musso, Filippo Mariano, Alberto Mella, Silvia Pinach, Roberto Gambino

**Affiliations:** ^1^ MECAU Department San Luigi Gonzaga Hospital, Orbassano Turin Italy; ^2^ Città della Salute e della Scienza, Department of Medical Sciences University of Turin Turin Italy

1

Metabolic dysfunction‐associated steatotic liver disease (MASLD) confers an increased risk of chronic kidney disease (CKD) [1] and is the most rapidly growing indication for simultaneous liver‐kidney transplantation, with poor renal outcomes [2]. Mechanisms promoting kidney dysfunction in MASLD are unclear and proposed MASLD medications did not show nephroprotection.

Nuclear Factor (NF)‐kB is a nuclear transcription factor at the crossroads of metabolic and nutritional inflammation: its activation in mononuclear cells (MNCs) drives their differentiation into a proinflammatory phenotype and recruits them to target organs: consistently, excessive NF‐kB activation in circulating MNCs has been involved in the pathogenesis of MASLD and CKD experimental models and in patients with diabetic nephropathy [3].

The postprandial phase, that is, the metabolic period encompassing the 6–8 h following a meal, during which digestion and absorption of nutrients occur, is emerging as a source of systemic low‐grade inflammation. As Western individuals currently consume several meals during the day, they spend most of the daytime in the postprandial phase: hypothesizing repetitive, meal‐induced NF‐kB‐mediated systemic inflammatory bouts may promote CKD in MASLD, we explored

(1) the relationship between postprandial NF‐kB activation in circulating MNCs and CKD in 85 biopsy‐proven MASLD patients (33 with metabolic‐dysfunction‐associated simple steatosis, MASL, and 52 with metabolic dysfunction‐associated steatohepatitis, MASH) (cross‐sectional cohort);

(2) the effect of changes in meal‐induced NF‐kB activation on renal outcomes in a subgroup of 52 MASLD patients treated with the enhanced curcumin formulation Meriva (a known NF‐kB inhibitor) or placebo in a randomized double‐blind [4] (longitudinal cohort). MASLD patients enrolled in the randomized trial repeated all baseline assessments, including liver biopsy and OTT, at end‐of‐treatment (EOT). Trial design, eligibility criteria, methods, and protocol are provided elsewhere [4].

All MASLD patients underwent a standardized 8‐h oral tolerance test (OTT) with thorough metabolic and inflammatory assessment, including measurement of NF‐kB activation in MNCs, and of plasma levels of Monocyte Chemoattractant Protein(MCP)‐1 and lumican, two downstream NF‐kB molecular targets, whose expression is upregulated by NF‐kB: MCP‐1 is a key chemokine mediating MNC recruitment to target organs, and lumican is a small leucine‐rich proteoglycan component of the extracellular matrix (ECM). Soluble, circulating lumican is an emerging mediator of lipotoxicity‐induced glomerular endothelial injury, ECM deposition and kidney injury in DN [5].

Subjects with and without CKD at EOT were compared for baseline values and for changes during follow‐up in the presence of CKD [defined according to Kidney Disease Improving Global Outcomes (KDIGO) guidelines as sustained (i.e., documented 3 month apart) eGFR < 90 mL/min/1.73 m^2^ and/or albumin excretion rate (AER) ≥30 mg/d)], and in absolute values and KDIGO categories of eGFR and AER (details in Supplementary Material).

Univariate and subsequent multivariate regression analyses were used to identify predictors of CKD presence (cross‐sectional cohort) and of CKD regression at EOT (longitudinal cohort); predictors of eGFR and AER were assessed using linear regression analysis.

Adjustments were made for treatment allocation and for variables a priori known to be associated with CKD (age, baseline eGFR and AER, diabetes, HbA1c, systolic blood pressure, baseline advanced hepatic fibrosis (stage F3‐4), LDL‐cholesterol, statin, angiotensin‐converting enzyme or angiotensin receptor blocker use. Additional covariates were chosen from variables which differed between MASLD‐CKD and MASLD without CKD at baseline. Sample size estimation, meal tolerance test, statistical analyses, and analytical assessments are detailed in Supplementary Material.

Use of GLP‐1 analogues or SGLT2 inhibitors was an exclusion criterion from the trial.

Consistent with existing literature [1, 2], 51 % of MASLD patients in the cross‐sectional cohort had CKD (Table S1): compared with MASLD patients without CKD, those with CKD showed higher postprandial NF‐kB activation in circulating MNCs, higher plasma levels of MCP‐1 and lumican, but similar fasting biochemical, clinical and metabolic profile (Table S1). Postprandial IAUC NF‐kB in MNCs was significantly associated with the presence of CKD and with eGFR and AER values even after adjustment for other covariates (Figure 1A; Table S2). Postprandial plasma MCP‐1 and Lumican peaked at 6 h, that is, 2 h after after plasma NF‐kB peak (Figure 1B).

**FIGURE 1:**
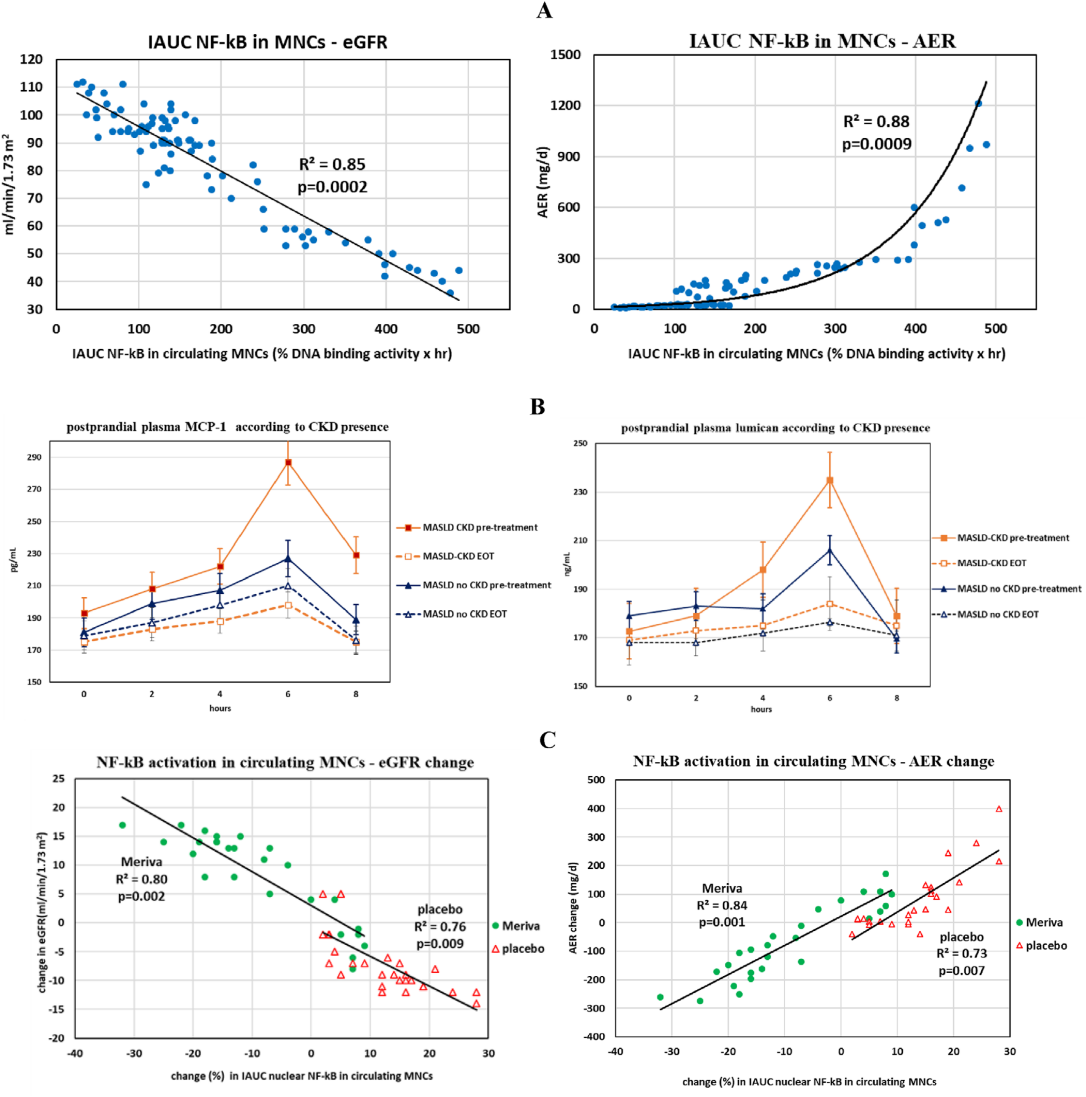
Meal tolerance test parameters. Data are presented as mean (SEM). (A) Correlation between postprandial NF‐kB activation in mononuclear cells (MNCs) and absolute values of eGFR and of AER in MASLD patients (cross‐sectional cohort, *n* = 85). (B) Plasma Monocyte Chemoattractant Protein (MCP)‐1 and Lumican during the standardized meal tolerance test. Patients (*n* = 52) were grouped according to the presence (*n* = 32) or absence (*n* = 20) of chronic kidney disease (CKD) at baseline. (C) Correlation between changes (%) in postprandial NF‐kB activation in MNCs and changes in absolute values of eGFR and of AER in MASH patients, grouped according to treatment arm (*n* = 52).

In the longitudinal cohort, Meriva treatment was associated with a significant decrease in postprandial NF‐kB activation in MNC**s** at EOT (Figure 1C): 20 (63%) of MASLD patients with CKD at baseline showed regression of renal disease, while 4 (20%) of patients without CKD at baseline developed CKD (Table S1).

The change in postprandial NF‐kB activation in MNCs was significantly associated with the change in CKD status at EOT and with changes in eGFR and AER values within each treatment group at univariate and multivariate analyses (Figure 1C, Table S2).

Notably, the slope of regression of changes in IAUC NF‐kB on eGFR and AER was similar across treatment groups (Figure 1C) suggesting that changes in postprandial NF‐kB activation are a key mediator of the renal effect in the two treatment arms (active + lifestyle intervention or placebo + lifestyle intervention).

A statistically significant relationship between changes in postprandial IAUC NF‐kB in MNCs and changes in plasma MCP‐1 (*r* = 0.76, *p* = 0.009) and lumican (*r* = 0.69, *p* = 0.011) at EOT was also observed.

In summary, the presence of CKD in our MASLD patients was associated with a prominent NF‐kB‐mediated proinflammatory activation of circulating MNCs in the postprandial phase. Pharmacological inhibition of NF‐kB ameliorated renal function and reversed CKD in MASLD.

Mechanisms underlying the abnormal meal‐induced NF‐kB activation require future investigation: while we ruled out excessive postprandial glucose and NEFA responses, which were similar in MASLD patients with and without CKD, potential triggers for NF‐kB activation in CKD include excessive intrinsic susceptibility of this transcription factor to nutrients or increased postprandial endotoxemia, which can by itself trigger NF‐kB activation.

Despite clear limitations, including the small sample size and the estimation of GFR rather than its direct measurement, these findings may have relevant therapeutic implications and point to NF‐kB inhibition as a feasible and effective therapeutic strategy for kidney disease in MASLD. The potential synergy of NF‐kB inhibitors with other drugs, including Sodium Glucose Cotransporter‐2 (SGLT2) inhibitors, which despite robust nephroprotection did not show hepatic histological benefit in MASLD [4] warrants also future evaluation.

## Author Contributions

**Giovanni Musso**: designed the trial, acquired and analyzed data, drafted the work, approved the final version, agreed to be accountable for all aspects of the work. He is the guarantor of the article. **Filippo Mariano**: gave substantial contributions to the study design and data acquisition, revised the work critically for important intellectual content, approved the final version to be published, agreed to be accountable for all aspects of the work. **Alberto Mella**: gave substantial contributions to the study design and data acquisition, revised the work critically for important intellectual content, approved the final version to be published, agreed to be accountable for all aspects of the work. **Silvia Pinach**: gave substantial contributions to the study design and data acquisition, revised the work critically for important intellectual content, approved the final version to be published, agreed to be accountable for all aspects of the work.**Roberto Gambino**: gave substantial contributions to the study design and data acquisition, revised the work critically for important intellectual content, approved the final version to be published, agreed to be accountable for all aspects of the work. All authors have read and approved the final manuscript.

## Ethics Statement

The study is funded by Indena Spa, Milan, is conducted in accordance with the ethical principles of the Declaration of Helsinki, was approved by A.O.U San Luigi Gonzaga hospital Ethics Committee (prot. N. 0008942) on May 25, 2018. All patients gave written informed consent to participate to the study.

## Conflicts of Interest

**Giovanni Musso**: no present or past financial or non‐financial competing interest to disclose. **Filippo Mariano**: no present or past financial or non‐financial competing interest to disclose. **Mella Alberto**: no present or past financial or non‐financial competing interest to disclose. **Silvia Pinach**: no present or past financial or non‐financial competing interest to disclose. **Roberto Gambino**: no present or past financial or non‐financial competing interest to disclose.

## Supporting information



Supporting Information

## Data Availability

Deidentified individual participant data will be made available with publication to anyone upon reasonable request to corresponding author Dr Giovanni Musso (e‐mail: giovanni_musso@yahoo.it), beginning after publication and up for 5 years.
