# Supplementary material for: Meal‐Induced NF‐kB Activation in Mononuclear Cells Triggers Lumican Secretion and Promotes Chronic Kidney Disease in Metabolic Dysfunction‐Associated Steatotic Liver Disease
Source: MedComm (2020). 2025 Aug 7;6(8):e70122. doi: 10.1002/mco2.70122 (PMC12331869; doi:10.1002/mco2.70122)
Supplement: Supplementary file 1 — Supporting Information [file MCO2-6-e70122-s001.docx]

**Supplementary material**

**Study design, data collection, and participants**

**Chronic Kidney Disease(CKD) definition.** CKD was defined according to KDIGO guidelines as sustained (i.e., documented on 2 occasions at least 3 months apart) reduction in eGFR<60 mL/min/1.73 m^2^ and/or sustained increase in AER≥30 mg/d. CKD regression was defined as sustained (i.e., documented 3 month apart, on both follow-up visit 7 and visit 8) eGFR≥60 mL/min/1.73 m^2^ **and** normalization of AER (i.e., <30 mg/d). eGFR was assessed from serum creatinine using the CKD-EPI (Chronic Kidney Disease Epidemiology Collaboration) equation, as recommended by KDIGO guidelines.

eGFR and urinary albumin excretion rate (AER) were classified according to Kidney Disease Improving Global Outcomes (KDIGO) categories.

**Standardized oral tolerance test composition.** Within 1 month from enrolment and 1 month from EOT liver biopsy, all patients underwent a standardized oral tolerance test^[[1]](#endnote-2)^ for a total energy content of 766 kcal. And the following composition: 75.3 g fat (55.6% saturated fatty acids, 29.6% monounsaturated fatty acids, 14.8% polyunsaturated fatty acids), 595 mg cholesterol, 9.8 g protein, 13 g carbohydrate. The meal was consumed over 5 minutes; subjects were kept fasting on the test morning, and strenuous activity will be forbidden. Participants were encouraged to avoid strenuous physical efforts and to follow their usual diet during the 24 hours preceding the test. Blood samples were drawn at 0 (baseline), 2, 4, 6, and 8 hours. Plasma total cholesterol (Chol), triglyceride (Tg), free fatty acids (FFA), glucose, insulin were measured by automated enzymatic methods.

**Sample size calculation.** There are no data on postprandial NF-kB responses in NASH with CKD. Based on previous data on postprandial lipid responses in NAFLD^[[2]](#endnote-3)^ ^[[3]](#endnote-4)^ ^[[4]](#endnote-5)^ assuming an effect size of 1.5(OR for CKD) across postprandial lipemia response quartiles and allowing for a 10% drop-out rate, at least 16 subjects per arm were needed to detect a significant (p<0.05) difference in CKD prevalence across triglyceride responses in NASH with a power of 80%.

**Analytical assessments**

**Nuclear Factor(NF)-kB activation in mononuclear cells (MNCs)**

Blood samples were collected in tubes containing Na-EDTA as an anticoagulant; 3.5 mL of the anticoagulated blood sample was carefully layered over 3.5 mL of the PMNL isolation medium (Robbins Scientific Corp, Sunnyvale, CA). Samples were centrifuged at 450 x *g* in a swing outrotor for 30 min at 22 °C. At the end of centrifugation, 2 bands separate out at the top of the red blood cell pellet. The top band consists of monocuclear leukocytes(MNCs), whereas the bottom band consists of polymorphumuclear leukocytes(PMNs). The MNC and PMN bands were harvested with a Pasteur pipette, repeatedly washed with Hank’s balanced salt solution, and reconstituted to a concentration of 4 x 10^5^ cells/mL in Hank’s balanced salt solution. This method yields> 95% pure PMN and MNC suspensions^[[5]](#endnote-6)^.

NF-kBp50/p65 transcription factor assay kit was purchased from Cayman Chemical (Ann Arbor MI, USA). The method detects specific transcription factor DNA binding activity in nuclear extracts and cell lysates. A specific double stranded DNA sequence containing the NF-kB response element is immobilized onto the bottom of wells of a 96 well-plate. NF-kB in nuclear or cytoplasmatic extract binds specifically to the NF-kB response element. NF-kBp50/65 is detected by addition of specific primary antibody directed against NF-kBp50/65. A secondary antibody conjugated to Horseradish peroxidase is added to provide a sensitive colorimetric readout at 450 nm.

**Monocyte chemoattractant protein-1(MCP-1), and lumican**

Monocyte chemoattractant protein-1(MCP-1) was measured using ELISA methods (Roche Modular system, Roche Ltd, Lewes, UK). Plasma lumican was analyzed by the ELISA technique using commercial kits (RayBiotech, USA). The sensitivity for MCP-1 assay was 1 pg/mL with an intra-assay variation coefficient < 10% and an interassay variation coefficient < 9%. The sensitivity for Lumican assay was 0.1 ng/mL with an intra-assay variation coefficient < 10% and an interassay variation coefficient < 12%^[[6]](#endnote-7),^^[[7]](#endnote-8),^^[[8]](#endnote-9)^.

**Statistical analyses**

Normality was evaluated by Shapiro-Wilk test and non-normal values were log-transformed for regression analysis. Fisher or chi‑square test were used to compare categorical variables, as appropriate. Differences across groups were analyzed by ANOVA and then by Bonferroni correction, when variables were normally distributed; otherwise the Kruskal-Wallis test, followed by the post hoc Dunn test, was used to compare nonparametric variables. All data were analyzed on an intention-to-treat basis.

For the meal tolerance test, the area under the curve(AUC) and incremental AUC(IAUC) of parameters were computed by the trapezoid method and compared with ANOVA repeated measures.

To adjust for multiple comparison testing, the Benjamini-Hochberg False Discovery Rate correction was applied to raw p-values in all comparisons; significance was set at an adjusted p-value threshold of 0.05.

Adjustments was made for treatment allocation and for variables *a priori* known to be associated with CKD progression (see main text). Continuous outcome measures were compared using linear regression, after log transformation of skewed parameters, We searched the best model fit among four predictive models (linear, exponential, logarithmic, binomial) using R-squared values. Adjustments were made for baseline values and allocated treatment (as model covariates, equivalent to ANCOVA), with multilevel modelling for key continuous outcome measures to account for repeated measures within each patient.

All analyses will be carried out with Easy R ver1.61, Saitama, Japan.

.

**Table S1.** Demographic, clinical, biochemical and histological characteristics of the cross-sectional cohort of MASLD patients (n=85) and of the longitudinal subgroup of MASLD patients(n=52) enrolled into the randomized trial with curcumin. Patients were grouped according to the presence of chronic kidney disease (CKD).

|  | **Cross-sectional cohort** | | **Longitudinal cohort** | | | |  |
| --- | --- | --- | --- | --- | --- | --- | --- |
|  | **MASLD**  **CKD**  **(n=43)** | **MASLD**  **no CKD**  **(n=42)** | **MASLD**  **CKD at baseline**  **(n=32)** | | **MASLD**  **no CKD at baseline**  **(n=20)** | |  |
|  |  |  | **Baseline** | **EOT** | **Baseline** | **EOT** | **P for between-group changes** |
| **Demographics** |  |  |  |  |  |  |  |
| **Age (years)** | 53 (10) | 52(11) | 55 (11) | 56 (11) | 53 (10) | 54 (10) | 0.638 |
| **Male** | 23 (53%) | 21 (50%) | 15 (47%) | 15(47%) | 11 (55%) | 11 (55%) | 0.489 |
| **Caucasian white race** | 40(93%) | 39(92%) | 30(94%) | 30(94%) | 19(95%) | 19(95%) | 0.528 |
| **Weight status**  **Obesity class I**  **Overweight** | 28 (66%)  15 (34%) | 28(67%)  14 (33%) | 23 (72%)  9 (28%) | 23 (72%)  9 (28%) | 14(70%)  6 (30%) | 14 (70%)  6 (30%) | 0.781  0.613 |
| **Current smoking** | 4 (9%) | 5 (12%) | 3 (9%) | 3 (9%) | 4 (20%) | 4 (20%) | 0.582 |
| **Comorbidities** |  |  |  |  |  |  |  |
| **Hypertension†** | 24 (56%) | 25(60%) | 18 (56%) | 18 (56%) | 12(60%) | 12 (60%) | 0.396 |
| **Type 2 diabetes** | 22(50%) | 19(45%) | 16(50%) | 16 (50%) | 9(45%) | 9 (45%) | 0.569 |
| **Hyperlipidaemia*** | 22 (50%) | 22 (52%) | 16 (50%) | 16(50%) | 12 (60%) | 12 (60%) | 0.478 |
| **Medications** |  |  |  |  |  |  |  |
| **Glucose-lowering**  **agents** |  |  |  |  |  |  |  |
| **Metformin** | 23 (53%) | 23 (55%) | 14 (44%) | 14 (44%) | 10 (50%) | 10 (50%) | 0.419 |
| **Insulin** | 14(33%) | 13(31%) | 9(28%) | 9 (28%) | 6(30%) | 7 (35%) | 0.573 |
| **Sulfonylurea** | 2 (5%) | 3 (6%) | 1 (3%) | 1 (3%) | 1 (5%) | 1 (5%) | 0.649 |
| **DPP-IV inhibitors** | 4 (10%) | 4 (10%) | 3 (9%) | 3 (9%) | 2 (10%) | 2 (10%) | 0.82 |
| **Anti-lipidaemic**  **Statins**  **PUFA**  **Fibrates** | 15 (35%)  3 (8%)  1 (2%) | 15 (36%)  2 (10%)  0 (0%) | 11 (34%)  3 (9%)  1 (3%) | 11 (34%)  3 (9%)  1 (3%) | 7 (35%)  2 (10%)  0 (0%) | 7 (35%)  2 (10%)  0 (0%) | 0.396  0.847  0.840 |
| **Anti-hypertensive** |  |  |  |  |  |  |  |
| **ACEI/ARB** | 21 (48%) | 22 (52%) | 16 (50%) | 16 (50%) | 11 (55%) | 11 (55%) | 0.893 |
| **Others** | 21(50%) | 21 (51%) | 15 (47%) | 15 (47%) | 11 (55%) | 11 (55%) | 0.528 |
| **Dietary habits** |  |  |  |  |  |  |  |
| **Total energy Intake (kcal/d)** | 2594(130) | 2573 (113) | 2501 (140) | 2398 (151) | 2569 (131) | 2402 (128) | 0.759 |
| **Kcal/kg BW/d** | 30 (2) | 30 (1) | 31 (1) | 31 (1) | 30 (1) | 30 (1) | 0.792 |
| **Alcohol (g/d)** | 4.1 (0.8) | 4.2(0.8) | 4.2 (0.7) | 4.0 (0.7) | 4.0(0.9) | 3.8 (0.8) | 0.713 |
| **Fat (% kcal/d)** | 34.9 (0.9) | 34.7 (0.8) | 35.1 (0.8) | 34.0 (0.9) | 34.2 (0.9) | 33.0 (1.0) | 0.594 |
| **CHO (% kcal/d)** | 49.8 (1.0) | 48.4(1.9) | 49.2 (1.1) | 48.2 (1.1) | 48.9(1.1) | 46.4 (1.0) | 0.573 |
| **Protein (% kcal/d)** | 15.6 (0.7) | 15.2 (0.9) | 16.1 (0.0) | 14.8 (0.7) | 15.1 (0.9) | 14.2 (0.9) | 0.429 |
| **SFA (% total fat)** | 34.5 (0.5) | 33.9 (0.8) | 34.9 (0.4) | 30.2 (0.4) | 33.4 (0.9) | 31.1 (0.7) | 0.712 |
| **MUFA(% total fat)** | 47.9 (1.2) | 47.1 (1.3) | 47.8 (1.3) | 48.9 (1.1) | 46.2 (1.1) | 47.2 (1.4) | 0.639 |
| **PUFA(% total fat)** | 11.4 (0.7) | 11.1 (0.8) | 12.1 (0.8) | 14.0 (0.9) | 11.3 (0.9) | 13.0 (1.2) | 0.498 |
| **Physical activity (PA) categories** |  |  |  |  |  |  |  |
| **Sedentary time(min/d)** | 812.6 (73.1) | 831.2 (90.3) | 809.8 (80.4) | 856.7 (91.2) | 838.4 (91.3) | 864.9 (89.9) | 0.769 |
| **Light PA (min/d)** | 188.9 (39.2) | 187.4 (37.9) | 171.8 (31.5) | 171.8 (31.5) | 193.4 (38.9) | 193.4 (38.9) | 0.813 |
| **Moderate PA(min/d)** | 99.5 (24.1) | 99.1 (12.1) | 98.6 (22.1) | 101.6 (18.1) | 99.7 (15.9) | 103.4 (14.7) | 0.849 |
| **Vigorous PA(min/d)** | 8.9 (6.1) | 9.0 (5.3) | 8.1 (7.3) | 8.9 (6.3) | 9.2 (7.2) | 10.0 (9.1) | 0.728 |
| **Metabolic parameters** |  |  |  |  |  |  |  |
| **Weight (kg)** | 101.6 (14.8) | 102.2 (19.5) | 103.9 (15.9) | 101.7 (19.4) | 102.7 (18.5) | 100.2 (15.3) | 0.413 |
| **BMI (kg/m²)** | 32.5(2.7) | 32.9 (3.1) | 33.4(3.1) | 33.1 (2.7) | 33.9 (3.2) | 33.3 (2.9) | 0.991 |
| **Waist circumference (cm)** | 103.1 (10.8) | 101.9 (10.7) | 107.3 (12.1) | 104.7 (10.9) | 106.1 (10.2) | 105.1 (10.2) | 0.739 |
| **HbA1c(%)** | 6.84 (0.91) | 6.51 (0.72) | 6.72 (0.51) | 6.01 (0.43)† | 6.68 (0.52) | 6.03 (0.43)† | 0.413 |
| **HOMA-IR** | 7.1 (5.3) | 7.2 (4.9) | 7.7 (4.9) | 6.4 (3.2)† | 7.9 (4.1) | 6.1 (3.1)† | 0.529 |
| **Total cholesterol (mg/dL)** | 191 (41) | 195 (43) | 196 (43) | 167(38)† | 199 (41) | 162(37)† | 0.492 |
| **LDL-C(mg/dL)** | 132 (28) | 135 (23) | 138 (24) | 122 (18)† | 136 (26) | 119 (19)† | 0.467 |
| **HDL-C(mg/dL)** | 38 (4) | 39 (4) | 39 (4) | 46 (4)† | 37 (4) | 44 (4)† | 0.518 |
| **Systolic blood pressure (mm Hg)** | 131 (11) | 132 (11) | 130 (13) | 127 (11) | 133 (12) | 130 (11) | 0.594 |
| **Diastolic blood pressure (mm Hg)** | 80 (12) | 77 (11) | 79 (11) | 78 (11) | 78 (9) | 75 (10) | 0.395 |
| **Renal function** |  |  |  |  |  |  |  |
| **Creatinine (mg/dL)** | 1.32 (0.28) ¶ | 0.81 (0.21) | 1.21 (0.20) ¶ | 0.89 (0.18)† | 0.87 (0.17) | 0.94 (0.28)† | 0.128 |
| **eGFR (mL/min/1.73 m2)** | 73(12) ¶ | 91(10) | 80(8) ¶ | 88(8)† | 92(10) | 97(10)† | 0.216 |
| **eGFR stage (ml/min/1.73 m^2^)** |  |  |  |  |  |  |  |
| **G1 (≥90 ml/min/1.73 m^2^)** | 0 (0%) | 42(100%) | 0 (0%) | 13 (41%)† | 20(100%) | 16(80%) | **0.009** |
| **G2(60-89)** | 22 (51%) | 0(42%) | 22 (69%) | 14 (44%) | 0(42%) | 3(15%) | 0.169 |
| **G3a(45-59)** | 16 (37%) | 0 (0%) | 10 (31%) | 5 (16%) | 0 (0%) | 1 (5%) | 0.314 |
| **G3b (30-44)** | 5 (12%) | 0 (0%) | 0 (0%) | 0 (0%) | 0 (0%) | 0 (0%) | 0.999 |
| **G4 (15-29)** | 0 (0%) | 0 (0%) | 0 (0%) | 0 (0%) | 0 (0%) | 0 (0%) | 0.999 |
| **G5(<15)** | 0 (0%) | 0 (0%) | 0 (0%) | 0 (0%) | 0 (0%) | 0 (0%) | 0.999 |
| **Albuminuria**  **(AER)(mg/day)** | 278(63) # | 20(8) | 192(55) # | 42(34)† | 20(9) | 61(22) |  |
| **A1 stage**  **(<30 mg/day )** | 0(0%) | 42(100%) | 0(0%) | 20(62%)† | 20(100%) | 16(80%) | 0.160 |
| **A2 stage (30-300)** | 38(88%) | 0(0%) | 32(100%) | 12(38%)† | 0(0%) | 4(20%) | 0.211 |
| **A3 stage (>300)** | 5(12%) | 0(0%) | 0(0%) | 0(0%) | 0(0%) | 0(0%) | 0.999 |
| **CKD (eGFR stage≥G2 and/or albuminuria stage≥A2)**  **n (%)** | 43 (100%)# | 0(0%) | 32 (100%)# | 12 (38%)‡ | 0(0%) | 4(20%) | **0.009** |
| **Liver function** |  |  |  |  |  |  |  |
| **Alanine aminotransferase (U/L)** | 89 (29) | 79 (37) | 81 (28) | 39 (22)† | 73 (32) | 37 (27)† | 0.213 |
| **Aspartate aminotransferase (U/L)** | 58(22) | 56 (28) | 57 (21) | 26 (18)† | 54 (24) | 32 (20)* | 0.393 |
| **γ-glutamyl transferase (U/L)** | 121 (67) | 112 (74) | 119 (68) | 73 (61)* | 102 (75) | 72 (59)* | 0.692 |
| **Alkaline phosphatase (U/L)** | 84 (29) | 82 (36) | 87 (25) | 78 (29) | 86 (38) | 74 (39) | 0.479 |
| **Total bilirubin (mg/dL)** | 0.80(0.22) | 0.62 (0.21) | 0.82 (0.24) | 0.73 (0.21) | 0.70 (0.19) | 0.60 (0.18) | 0.394 |
| **Albumin (g/dL)** | 3.6(0.5) | 3.7(0.6) | 3.7 (0.6) | 3.9 (0.6) | 3.6(0.5) | 4.0 (0.5) | 0.529 |
| **Noninvasive markers of liver disease severity** |  |  |  |  |  |  |  |
| **Sonographic Hepato/Renal ratio** | 2.71(0.71) | 2.68(0.63) | 2.62(0.74) | 1.12(0.47)† | 2.60(0.60) | 1.23(0.58) | 0.397 |
| **FIB-4** | 1.82(0.28) | 1.96(0.31) | 2.03(0.36) | 1.57(0.39)* | 1.97(0.33) | 1.56(0.41)* | 0.479 |
| **Liver histology** |  |  |  |  |  |  |  |
| **MASH** | 32 (74%) | 20 (48%) | 32 (100%) | 21 (66%) | 20 (100%) | 11 (55%) | 0.460 |
| **NAFLD activity score (0–8)** | 6.5 (0·9) ¶ | 5.3 (0·6) | 7.7 (0·8) ¶ | 4.2 (0·6)‡ | 5.2 (0·9) | 3.6 (0·9)‡ | 0.129 |
| **Hepatocyte ballooning score (0–2)** | 1.6 (0.5) | 1.5 (0.5) | 1.7 (0.4) | 1.3 (0.3)* | 1.4 (0.5) | 1.0 (0.3)* | 0.314 |
| **Steatosis score (0–3)** | 2.0 (0.6) | 2.410.6) | 2.2 (0.7) | 1.2 (0.7)† | 2.4(0.5) | 1.3(0.5)† | 0.485 |
| **Lobular inflammation score (0–3)** | 2.4 (0.9) | 1.4 (0.5) | 1.9 (0.6) | 1.6 (0.5)† | 1.5 (0.4) | 1.1 (0.4)† | 0.759 |
| **Kleiner fibrosis stage (F0–F4)** | 1.8 (0.9) | 1.7 (1.0) | 2.0 (1.3) | 1.9 (1.3) | 1.8 (0.9) | 1.7(0.8) | 0.748 |
| **Kleiner fibrosis stages (F0-F4)**  **F0**  **F1**  **F2**  **F3**  **F4** | 1(3%)  8 (19%)  20(46%)  11 (26%)  2 (6%) | 2(5%)  15(35%)  12(30%)  11(25%)  2 (5%) | 1 (3%)  6 (19%)  15(47%)  9 (28%)  2 (6%) | 3 (9%)  11 (34%)  9(28%)  7 (22%)  3 (9%) | 1 (5%)  7 (35%)  6(30%)  4 (25%)  1 (5%) | 1 (5%)  6 (30%)  4(20%)  4 (20%)  2 (10%) | 0.397  0.447  0.693  0.559  0.692 |
| **Clinically significant fibrosis (stage F2-4) n (%)** | 26 (60%) ¶ | 23 (55%) | 26 (81%) ¶ | 19 (73%) | 11 (55%) | 10 (50%) | 0.394 |
| **Advanced fibrosis (stage 3-4) n (%)** | 13 (30%) | 13 (31%) | 11 (34%) | 10 (31%) | 5 (25%) | 6 (30%) | 0.893 |
| **Oral tolerance test** |  |  |  |  |  |  |  |
| **Fasting Tg (mg/dL)** | 154 (30) | 138 (32) | 151 (33) | 134 (35) | 143 (29) | 122 (24) | 0.285 |
| **IAUC Tg**  **(mg/dL x hr)** | 199(61) | 186(26) | 197(21) | 148(17)* | 182(24) | 132(21)* | 0.297 |
| **Fasting NEFA (mmol/L)** | 0.53(0.22) | 0.56 (0.20) | 0.56 (0.24) | 0.46 (0.24) | 0.52 (0.21) | 0.41 (0.21) | 0.313 |
| **IAUC NEFA**  **(mMol/L x hr)** | 1.99(0.38) | 1.84(0.42) | 1.97(0.43) | 1.11(0.38) | 1.81(0.48) | 1.03(0.33) | 0.339 |
| **Fasting LDL-C (mg/dL)** | 135 (22) | 138 (21) | 138 (24) | 122 (18)* | 136 (26) | 119 (19)* | 0.461 |
| **IAUC LDL-C**  **(mg/dL x hr)** | 41(22) | 44(24) | 43(22) | 32(18) | 48(24) | 36(22) | 0.398 |
| **Fasting plasma glucose (mg/dL)** | 114 (22) | 113 (16) | 118 (28) | 110 (22) | 117 (22) | 109 (20) | 0.397 |
| **IAUC plasma glucose (mg/dL x hr)** | 123(42) | 129(36) | 124(49) | 114(41) | 122(38) | 119(32) | 0.350 |
| **Fasting insulin (uU/mL)** | 24.9(10.1) | 22.8 (11.3) | 26.3 (11.4) | 18.1 (10.1) | 24.8 (12.1) | 16.1 (9.8) | 0.279 |
| **IAUC insulin (uU/mL x hr)** | 35(36) | 32(39) | 39(38) | 32(31( | 38(42) | 38(27) | 0.572 |
| **Fasting MCP-1**  **(pg/mL x hr)** | 187(33) | 188(25) | 193(32) | 179(24) | 181(28( | 175(22) | 0.312 |
| **IAUC MCP-1**  **(pg/mL x hr)** | 537(41) # | 372(41) | 541(44) # | 271(31)‡ | 377(39) | 263(32)† | 0.485 |
| **Fasting Lumican (ng/mL)** | 172 (31) | 178 (28) | 179(26) | 171(25) | 188 (28) | 177(33) | 0.514 |
| **IAUC Lumican (ng/mL x hr)** | 365(41) | 221(39) | 373(46) | 212(26) | 228/31) | 187(24) | 0.183 |
| **nuclear NF-κB in MNCs**  **(%DNA binding activity)** | 30(4) | 28(5) | 28(2) | 26(2) | 26(2) | 25(2) | 0.691 |
| **IAUC nuclear NF-κB in MNCs**  **(%DNA binding activity x hr)** | 328(39) # | 161(39) | 316(48) # | 76(13)‡ | 142(36) | 58(11)‡ | 0.198 |

Data are n (%) or mean (SEM).

Abbreviations: BMI: Body-mass index; C: cholesterol; DPP-IV: dipeptidy peptidase (DPP) IV inhibitors; HOMA-IR=homeostasis model assessment of insulin resistance; IHC: Immmunohistochemistry; MCP: monocyte chemoattractant protein; MNCs: mononuclear cells: NF-kB: nuclear factor-kB; Tg: triglyceride.

* p<0.05 vs. baseline within the same group (MASLD-CKD or MASLD without CKD)

† p<0.01 vs. baseline within the same group

‡ p<0.001 vs. baseline within the same group

¶ p<0.01 vs. MASLD without CKD at baseline

# p<0.001 vs. MASLD without CKD at baseline

**Hyperlipidaemia** was defined as recorded in the past medical history, as receiving lipid-lowering drugs (eg, statin, fibrate, ezetimibe), or both.

**Hypertension** was defined as recorded in the past medical history, as receiving an anti–hypertensive drug, or both. LDL concentration was calculated using the Friedwald formula.

**Non-alcoholic fatty liver disease (NAFLD) activity score** is the algebric sum of steatosis score, lobular inflammation score and hepatocyte ballooning score

**Table S2:** Multivariate regression models for the presence of CKD at baseline (cross-sectional cohort) and at the end-of-treatment (EOT)(longitudinal cohort).

| **Outcome: presence of CKD in MASLD**  **(cross-sectional cohort, n=85)** |  |  |
| --- | --- | --- |
| **Model** | **Odds ratio (95% CI)** | **p-value** |
| Baseline hepatic fibrosis (F3-4) | 1.62 (0.71, 8.13) | 0.199 |
| Baseline NAFLD Activity Score(NAS) | 1.45 (0.73-8.49) | 0.413 |
| Diabetes (present) | 1.89 (0.78, 4.49) | 0.317 |
| Obesity (present) | 1.62 (0.38, 4.73) | 0.513 |
| Dyslipidemia (present) | 1.11 (0.32-10.51) | 0.793 |
| Hypertension (present) | 1.32(0.12-6.95) | 0.711 |
| ACEIs/ARBs use | 0.91 (0.82, 2.39) | 0.216 |
| IAUC NF-kB activation in circulating MNCs (highest tertile) | 3.71 (2.18, 5.39) | **0.004** |
| IAUC plasma MCP-1 (highest tertile) | 2.12 (0.81-4.39) | 0.269 |
| IAUC lumican (highest tertile) | 2.57 (0.90-4.53) | 0.186 |
| **Outcome: CKD at EOT in MASLD**  **(longitudinal cohort, n=52)** |  |  |
| **Model** | **Odds ratio (95% CI)** | **p-value** |
| Age | 1.03 (0.78-4.39) | 0.523 |
| Treatment allocation (curcumin) | 4.12 (0.80-12.21) | 0.106 |
| HbA1c change | 0.87 (0.19-3.98) | 0.571 |
| eGFR at baseline | 0.58(0.12-4.81) | 0.729 |
| AER at baseline | 0.49(0.18-6.33) | 0.481 |
| Systolic BP change | 0.81 (0.14-3.97) | 0.512 |
| LDL-cholesterol change | 1.01 (0.39-4.26) | 0.720 |
| IAUC NF-kB activation in circulating MNCs change (%) | 4.39 (1.21-5.13) | **0.009** |
| IAUC plasma MCP-1 change | 3.98(0.72-3.81) | 0.112 |
| IAUC plasma lumican change | 2.13(0.83-5.18) | 0.392 |
| **Outcome: eGFR change at EOT**  **(longitudinal cohort, n=52)** |  |  |
| **Model** | **(SE)** | **p-value** |
| Age | -0.199 (0.127) | 0.210 |
| Treatment allocation (curcumin) | 0.231(0.138) | 0.227 |
| HbA1c change | 0.214(0.192) | 0.613 |
| eGFR at baseline | -0.332(0.003) | **0.035** |
| AER at baseline | -0.201(0.172) | 0.308 |
| Systolic BP change | -0.199(0.127) | 0.210 |
| LDL-cholesterol change | -0.288(0.139) | 0.407 |
| IAUC NF-kB activation in circulating MNCs change(%) | -0.510(0.008) | **0.006** |
| IAUC plasma MCP-1 change | -0.324(0.009) | 0.073 |
| IAUC plasma lumican change | -0.281(0.001) | 0.319 |
| **Outcome: AFR change at EOT**  **(longitudinal cohort, n=52)** |  |  |
| **Model** | **(SE)** | **p-value** |
| Age | -0.102 (0.181) | 0.729 |
| Treatment allocation (curcumin) | 0.237(0.132) | 0.124 |
| HbA1c change | 0.187(0.123) | 0.713 |
| eGFR at baseline | -0.331(0.006) | 0.259 |
| AER at baseline | -0.383(0.176) | **0.039** |
| Systolic BP change | -0.102(0.194) | 0.713 |
| LDL-cholesterol change | -0.179(0.138) | 0.692 |
| IAUC NF-kB activation in circulating MNCs change(%) | -0.503(0.008) | **0.007** |
| IAUC plasma MCP-1 change | -0.329(0.012) | 0.112 |
| IAUC plasma lumican change | -0.252(0.139) | 0.394 |

1. Kolovou GD, Watts GF, Mikhailidis DP, et al Postprandial Hypertriglyceridaemia Revisited in the Era of Non-Fasting Lipid Profile Testing: A 2019 Expert Panel Statement, Main Text. Curr Vasc Pharmacol. 2019;17:498-514. [↑](#endnote-ref-2)
2. Musso G, Cassader M, De Michieli F, Rosina F, Orlandi F, Gambino R. Nonalcoholic steatohepatitis versus steatosis: adipose tissue insulin resistance and dysfunctional response to fat ingestion predict liver injury and altered glucose and lipoprotein metabolism. Hepatology. 2012;56: 933-42 [↑](#endnote-ref-3)
3. Grandt J, Jensen AH, Werge MP, et al. Postprandial dysfunction in fatty liver disease. Physiol Rep. 2023;11:e15653. [↑](#endnote-ref-4)
4. Li X, Zheng K, Liu L, et al. Relationship of postprandial fibroblast growth factor 21 with lipids, inflammation and metabolic dysfunction-associated fatty liver disease during oral fat tolerance test. Front Endocrinol (Lausanne). 2024;15: 1343853. [↑](#endnote-ref-5)
5. [?]Ghanim H, Abuaysheh S, Sia CL. Increase in plasma endotoxin concentrations and the expression of Toll-like receptors and suppressor of cytokine signaling-3 in mononuclear cells following a high-fat high-carbohydrate meal: implications for insulin resistance. Diabetes Care 2009;32:2281–2287 [↑](#endnote-ref-6)
6. Decaris ML, Li KW, Emson CL, et al. Identifying nonalcoholic fatty liver disease patients with active fibrosis by measuring extracellular matrix remodeling rates in tissue and blood. Hepatology. 2017 ;65:78-88. [↑](#endnote-ref-7)
7. Krishnan A, Li X, Kao WY, et al. Lumican, an extracellular matrix proteoglycan, is a novel requisite for hepatic fibrosis. Lab Invest. 2012;92:1712-25. [↑](#endnote-ref-8)
8. Charlton M, Viker K, Krishnan A, et al. Differential expression of lumican and fatty acid binding protein-1: new insights into the histologic spectrum of nonalcoholic fatty liver disease. Hepatology. 2009;49:1375-84 [↑](#endnote-ref-9)
